# Comparison of solution-based exome capture methods for next generation sequencing

**DOI:** 10.1186/gb-2011-12-9-r94

**Published:** 2011-09-28

**Authors:** Anna-Maija Sulonen, Pekka Ellonen, Henrikki Almusa, Maija Lepistö, Samuli Eldfors, Sari Hannula, Timo Miettinen, Henna Tyynismaa, Perttu Salo, Caroline Heckman, Heikki Joensuu, Taneli Raivio, Anu Suomalainen, Janna Saarela

**Affiliations:** 1 Institute for Molecular Medicine Finland (FIMM), University of Helsinki, Biomedicum Helsinki 2U, Tukholmankatu 8, 00290 Helsinki, Finland; 2 Unit of Public Health Genomics, National Institute for Health and Welfare, Biomedicum Helsinki, Haartmaninkatu 8, 00290 Helsinki, Finland; 3 Research Programs Unit, Molecular Neurology, Biomedicum-Helsinki, University of Helsinki, Haartmaninkatu 8, 00290 Helsinki, Finland; 4 Department of Oncology, Helsinki University Central Hospital (HUCH), Haartmaninkatu 4, 00290 Helsinki, Finland; 5 Institute of Biomedicine, Department of Physiology, University of Helsinki, Haartmaninkatu 8, 00290 Helsinki, Finland; 6 Children's Hospital, Helsinki University Central Hospital (HUCH), Stenbäckinkatu 11, 00290 Helsinki, Finland

## Abstract

**Background:**

Techniques enabling targeted re-sequencing of the protein coding sequences of the human genome on next generation sequencing instruments are of great interest. We conducted a systematic comparison of the solution-based exome capture kits provided by Agilent and Roche NimbleGen. A control DNA sample was captured with all four capture methods and prepared for Illumina GAII sequencing. Sequence data from additional samples prepared with the same protocols were also used in the comparison.

**Results:**

We developed a bioinformatics pipeline for quality control, short read alignment, variant identification and annotation of the sequence data. In our analysis, a larger percentage of the high quality reads from the NimbleGen captures than from the Agilent captures aligned to the capture target regions. High GC content of the target sequence was associated with poor capture success in all exome enrichment methods. Comparison of mean allele balances for heterozygous variants indicated a tendency to have more reference bases than variant bases in the heterozygous variant positions within the target regions in all methods. There was virtually no difference in the genotype concordance compared to genotypes derived from SNP arrays. A minimum of 11× coverage was required to make a heterozygote genotype call with 99% accuracy when compared to common SNPs on genome-wide association arrays.

**Conclusions:**

Libraries captured with NimbleGen kits aligned more accurately to the target regions. The updated NimbleGen kit most efficiently covered the exome with a minimum coverage of 20×, yet none of the kits captured all the Consensus Coding Sequence annotated exons.

## Background

The capacity of DNA sequencing has increased exponentially in the past few years. Sequencing of a whole human genome, which previously took years and cost millions of dollars, can now be achieved in weeks [1-3]. However, as pricing of whole-genome sequencing has not yet reached the US$1000 range, methods for focusing on the most informative and well-annotated regions - the protein coding sequences - of the genome have been developed.

Albert *et al. *[4] introduced a method to enrich genomic loci for next generation re-sequencing using Roche NimbleGen oligonucleotide arrays in 2007, just prior to Hodges and collaborators [5], who applied the arrays to capture the full human exome. Since then, methods requiring less hands-on work and a smaller amount of input DNA have been under great demand. A solution-based oligonucleotide hybridization and capture method based on Agilent's biotinylated RNA baits was described by Gnirke *et al. *in 2009 [6]. Agilent SureSelect Human All Exon capture was the first commercial sample preparation kit on the market utilizing this technique, soon followed by Roche NimbleGen with the SeqCap EZ Exome capture system [7]. The first authors demonstrating the kits' capability to identify genetic causes of disease were Hoischen *et al. *(Agilent SureSelect) [8] and Harbour *et al. *(NimbleGen SeqCap) [9] in 2010. To date, exome sequencing verges on being the standard approach in studies of monogenic disorders, with increasing interest in studies of more complex diseases as well. The question often asked from a sequencing core laboratory is thus: 'Which exome capture method should I use?'

The sample preparation protocols for the methods are highly similar; the greatest differences are in the capture probes used, as Agilent uses 120-bp long RNA baits, whereas NimbleGen uses 60- to 90-bp DNA probes. Furthermore, Agilent SureSelect requires only a 24-hour hybridization, whereas NimbleGen recommends an up to 72-hour incubation. No systematic comparison of the performance of these methods has yet been published despite notable differences in probe design, which could significantly affect hybridization sensitivity and specificity and thus the kits' ability to identify genetic variation.

Here we describe a comprehensive comparison of the first solution-based whole exome capture methods on the market; Agilent SureSelect Human All Exon and its updated version Human All Exon 50 Mb, and Roche NimbleGen SeqCap EZ Exome and its updated version SeqCap EZ v2.0. We have compared pairwise the performance of the first versions and the updated versions of these methods on capturing the targeted regions and exons of the Consensus Coding Sequence (CCDS) project, their ability to identify and genotype known and novel single nucleotide variants (SNVs) and to capture small insertion-deletion (indel) variants. In addition, we present our variant-calling pipeline (VCP) that we used to analyze the data.

## Results

### Capture designs

**Figure 1:**
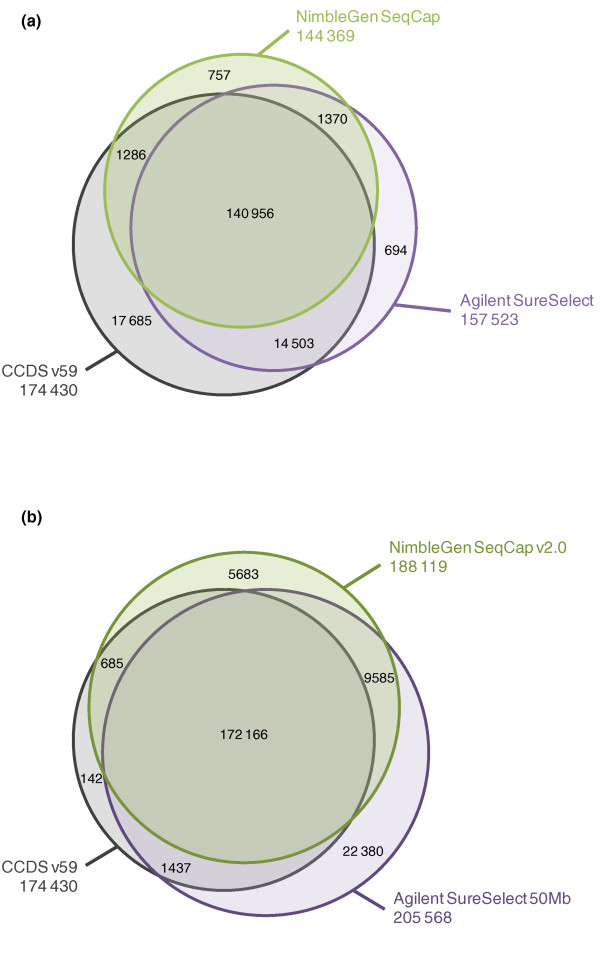
**Comparison of the probe designs of the exome capture kits against CCDS exon annotations**. **(a, b) **Given are the numbers of CCDS exon regions, common target regions outside CCDS annotations and the regions covered individually by the Agilent SureSelect and NimbleGen SeqCap sequence capture kits (a) and the Agilent SureSelect 50 Mb and NimbleGen SeqCap v2.0 sequence capture kits (b). Regions of interest are defined as merged genomic positions regardless of their strandedness, which overlap with the kit in question. Sizes of the spheres are proportional to the number of targeted regions in the kit. Total numbers of targeted regions are given under the name of each sphere.

The probe designs of Agilent SureSelect Human All Exon capture kits (later referred to as Agilent SureSelect and Agilent SureSelect 50 Mb) and NimbleGen SeqCap EZ Exome capture kits (later referred to as NimbleGen SeqCap and NimbleGen SeqCap v2.0) are compared in Figure 1 and Additional file 1 with the CCDS project exons [10] and the known exons from the UCSC Genome Browser [11]. Agilent SureSelect included 346,500 and SureSelect 50 Mb 635,250 RNA probes of 120 bp in length targeting altogether 37.6 Mb and 51.6 Mb of sequence, respectively. Both NimbleGen SeqCap kits had approximately 2.1 million DNA probes varying from 60 bp to 90 bp, covering 33.9 Mb in the SeqCap kit and 44.0 Mb in the SeqCap v2.0 kit in total. The Agilent SureSelect design targeted about 13,300 CCDS exon regions (21,785 individual exons) more than the NimbleGen SeqCap design (Figure 1a and Table 1). With the updated exome capture kits, Agilent SureSelect 50 Mb targeted 752 CCDS exon regions more than NimblGen SeqCap v2.0, but altogether it had 17,449 targeted regions and 1,736 individual CCDS exons more than the latter (Figure 1b). All of the exome capture kits targeted nearly 80% of all microRNAs (miRNAs) in miRBase v.15 at the minimum. The GC content of the probe designs of both vendors was lower than that of the whole CCDS exon regions (Table 1).Only Agilent avoided repetitive regions in their probe design **(**RepeatMasker April 2009 freeze). Neither of the companies had adjusted their probe designs according to the copy number variable sequences (Database of Genomic Variants, March 2010 freeze).

**Table 1:** Capture probe designs of the compared exome capture kits

Exome capture method	Probes	Base pairs covered (kb)	CCDS exons targeted^a^	Complete CCDS transcripts targeted^b^	miRNAs targeted^c^	Mean GC content of the target regions^d^	Percentage of base pairs in repeats^e^	Percentage of base pairs in CNVs^f^
Agilent SureSelect	347 k	37,627	274,264	20,699	646	50.56%	0.2%	34.5%
Agilent SureSelect 50 Mb	635 k	51,647	300,040	23,031	669	50.56%	0.8%	38.3%
NimbleGen SeqCap	2.1 M	33,931	252,479	18,865	559	50.45%	1.3%	33.9%
NimbleGen SeqCap v2.0	2.1 M	44,007	298,304	23,028	686	50.34%	2.1%	35.3%

^a^There are 301,082 exons annotated in total in CCDS from Ensembl v59. ^b^All CCDS annotated exons of a transcript are required to be included in the capture target region. There are 23,634 transcripts in total in CCDS from Ensembl v59. ^c^There are 712 miRNAs in total in miRBase v.15. ^d^The mean GC content for all CCDS annotated exon regions is 52.12%. ^e^RepeatMasker, April 2009 freeze. ^f^Database of Genomic Variants, March 2010 freeze. CNV, copy number variation; M, million.

### Variant-calling pipeline

**Figure 2:**
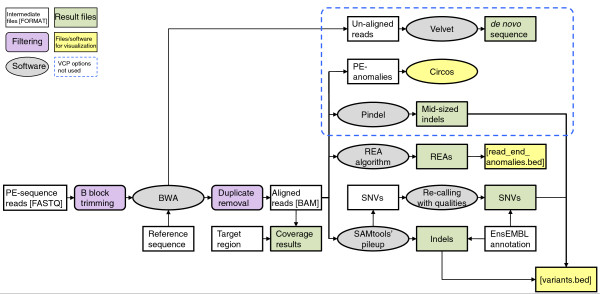
**Overview of the variant calling pipeline**. VCP consists of sequence analysis software and in-house built algorithms, and its output gives a wide variety of sequencing results. Sequence reads are first filtered for quality. Sequence alignment is then performed with BWA, followed by duplicate removal, variant calling with SAMtools' pileup and in-house developed algorithms for SNV calling with qualities and REA calling. File transformation programs are used to convert different file formats between the software. White boxes, files and intermediate data; purple boxes, filtering steps; grey ellipses, software and algorithms; green boxes, final VCP output; yellow boxes, files for data visualization; area circled with blue dashed line, VCP analysis options not used in this study. PE, paired end.

A bioinformatics pipeline for quality control, short read alignment, variant identification and annotation (named VCP) was developed for the sequence data analyses. Existing software were combined with in-house developed algorithms and file transformation programs to establish an analysis pipeline with simple input files, minimum hands-on work with the intermediate data and an extensive variety of sequencing results for all kinds of next-generation DNA sequencing experiments. In the VCP, sequence reads in FASTQ format were first filtered for quality. Sequence alignment was then performed with Burrows-Wheeler Aligner (BWA) [12], followed by duplicate removal. Variant calling was done with SAMtools' pileup [13], with an in-house developed algorithm using allele qualities for SNV calling, and with read end anomaly (REA) calling (see the 'Computational methods' section for details). In addition to tabular formats, result files were given in formats applicable for visualization in the Integrative Genomics Viewer [14] or other sequence alignment visualization interfaces. An overview of the VCP is given in Figure 2. In addition, identification of indels with Pindel [15], visualization of anomalously mapping paired-end (PE) reads with Circos [16] and *de novo *alignment of un-aligned reads with Velvet [17] were included in the VCP, but these analysis options were not used in this study.

### Sequence alignment

We obtained 4.7 Gb of high quality sequence with Agilent SureSelect and 5.1 Gb with NimbleGen SeqCap, of which 81.4% (Agilent) and 84.4% (NimbleGen) mapped to the human reference sequence hg19 (GRCh37). For the updated kits the obtained sequences were 5.6 Gb for the Agilent SureSelect 50 Mb and 7.0 Gb for the NimbleGen SeqCap v2.0, and the percentage of reads mapping to the reference was 94.2% (Agilent) and 75.3% (NimbleGen). Table 2 presents the sequencing and mapping statistics for individual lanes as well as the mean sequencing and mapping values from the 25 additional exome samples (see Material and methods for details). The additional exome samples were aligned only against the reference genome and the capture target region (CTR) of the kit in question, so only these numbers are shown. In general, sequencing reads from the NimbleGen exome capture kits had more duplicated read pairs than the Agilent kits. On average, 14.7% of high quality reads were duplicated in NimbleGen SeqCap versus 10.0% that were duplicated in Agilent SureSelect (*P *> 0.05) and 23.3% were duplicated in SeqCap v2.0 versus 7.3% that were duplicated in SureSelect 50 Mb (*P *= 0.002). However, the alignment of the sequence reads to the CTR was more precise using the NimbleGen kits and resulted in a greater amount of deeply sequenced (≥ 20×) base pairs in the target regions of interest. On average, 61.8% of high quality reads aligned to the CTR and 78.8% of the CTR base pairs were covered with a minimum sequencing depth of 20× with NimbleGen SeqCap versus 51.7% of reads that aligned to the CTR and 69.4% of base pairs that were covered with ≥ 20× with Agilent SureSelect (*P *= 0.031 and *P *= 5.7 × 10^-4^, respectively). For the updated kits, 54.0% of the reads aligned to the CTR and 81.2% of base pairs covered with ≥ 20× with SeqCap v2.0 versus 45.1% of reads that aligned to the CTR and 60.3% of base pairs that were covered with ≥ 20× with SureSelect 50 Mb (*P *= 0.009 and *P *= 5.1 × 10^-5^, respectively).

**Table 2:** Statistics of the sequencing lanes for the control I sample and mean values for the additional samples

							Percentage of base pairs in the target region covered ≥ 20×^b^			
							
Exome capture method	Read length (bp)	Number of high quality reads^a^	Mb of sequence	Percentage of reads removed in duplicate removal	Percentage of high quality reads aligned to hg19	Percentage of high quality reads aligned to CTR	CTR	CTR + flank	CCDS	Common
Agilent SureSelect										
Lane 1	60	32,943,000	1,980	6.45%	90.27%	54.71%	45.31%	29.05%	42.04%	46.57%
Lane 2	82	57,259,000	4,700	9.24%	81.42%	51.50%	60.84%	46.87%	54.54%	61.82%
Combined		90,202,000	6,670	8.22%	84.65%	52.67%	68.15%	55.3%	60.98%	69.2%
										
Agilent SureSelect 50 Mb										
Lane 1	82	41,871,000	3,430	5.23%	93.59%	42.96%	45.66%	33.62%	47.04%	44.71%
Lane 2	82	56,407,000	4,630	6.15%	92.37%	42.25%	53.72%	42.83%	54.54%	53.81%
Conditionally combined^c^	82	67,755,000	5,560	4.44%	94.15%	43.17%	60.25%	50.05%	60.95%	60.82%
										
NimbleGen SeqCap										
Lane 1	60	33,518,000	2,010	8.98%	90.79%	73.57%	56.96%	38.24%	44.78%	59.18%
Lane 2	82	62,141,000	5,100	14.92%	84.42%	71.27%	75.41%	57.52%	59.69%	77.1%
Combined		95,659,000	7,110	12.84%	86.65%	72.08%	82.33%	66.65%	65.45%	83.74%
										
NimbleGen SeqCap v2.0										
Lane 1	82^d^	85,072,000	6,980	24.48%	75.27%	51.22%	79.99%	70.84%	77.55%	81.1%
										
Mean for the additional samples^e^										
Agilent SureSelect (*n *= 2)	82	71,201,000	5,840	10.37%	88.72%	51.84%	73.63%	-	-	-
Agilent SureSelect 50 Mb (*n *= 2)	82	67,089,000	5,500	8.65%	90.41%	46.03%	60.30%	-	-	-
NimbleGen SeqCap (*n *= 19)	82	67,626,000	5,550	14.66%	81.98%	61.25%	78.94%	-	-	-
NimbleGen SeqCap v2.0 (*n *= 2)	82^f^	70,638,000	5,790	22.76%	76.97%	55.35%	81.82%	-	-	-

^a^Number of reads after B block trimming. ^b^Target region abbreviations: CTR, own capture target region of the kit; CTR + flank, own capture target region ± 100 bp; CCDS, exon annotated regions from CCDS, Ensembl v59; Common, regions captured by all the kits in comparison. ^c^Data from the sequencing lanes combined and randomly down-sampled to meet comparable read amounts after filtering. ^d^Sequenced with 100 bp, reads trimmed to 82 bp prior to any other action. ^e^The additional exome samples were aligned only against the whole genome and own capture target region. ^f^Sequenced with 110 bp, reads trimmed to 82 bp prior to any other action.

**Figure 3:**
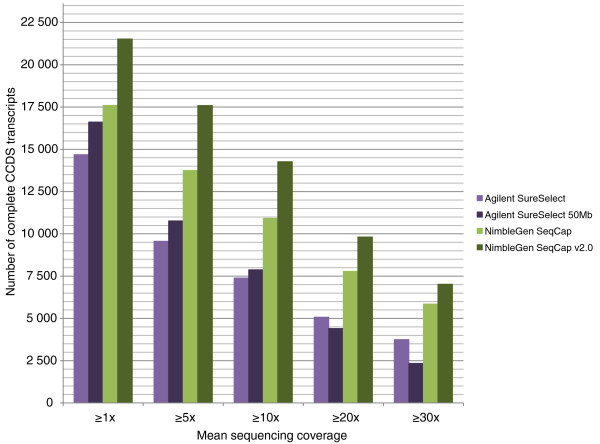
**Number of fully covered CCDS transcripts with different minimum coverage thresholds**. For each exon, median coverage was calculated as the sum of sequencing coverage on every nucleotide in the exon divided by the length of the exon. If all the annotated exons of a transcript had a median coverage above a given threshold, the transcript was considered to be completely covered. The number of all CCDS transcripts is 23,634.

When mutations underlying monogenic disorders are searched for with whole exome sequencing, every missed exon causes a potential need for further PCR and Sanger sequencing experiments. We thus wanted to evaluate the exome capture kits' capability to capture all coding sequences of the human genome by assessing how many complete CCDS transcripts (that is, having captured all the annotated exons from the transcript) the kits actually captured in the control I sample. The number of complete transcripts captured with a minimum coverage of 20× was 5,074 (24.5% of all targeted complete transcripts in the CTR) for Agilent SureSelect, 4,407 (19.1% of targeted transcripts) for Agilent SureSelect 50 Mb, 7,781 (41.3% of targeted transcripts) for NimbleGen SeqCap and 9,818 (42.6% of targeted transcripts) for NimbleGen SeqCap v2.0. The respective percentages of the captured, targeted individual exons were 65.8% (55.8% of all annotated exons), 62.0% (57.6%), 83.4% (65.1%) and 85.3% (78.7%). Figure 3 shows the numbers of complete transcripts captured with each exome capture method with different minimum mean thresholds. Individual CCDS exons targeted by the methods and their capture successes in the control I sample are given in Additional files 2 to 5.

We examined in detail the target regions that had poor capture success in the control I sample. GC content and mapability were determined for the regions in each method's CTR, and the mean values were compared between regions with mean sequencing depths of 0×, < 10×, ≥ 10× and ≥ 20×. High GC content was found to be associated with poor capture success in all exome enrichment methods. Table 3 shows the mean GC content for targets divided in groups according to mean sequencing coverage. We found no correlation with the sequencing depth and mapability. To compare poorly and well captured regions between the different capture kits, GC content and mapability were determined for the common regions that were equally targeted for capture in all kits. Regions with poor capture success in one method (0×) and reasonable capture success in another method (≥ 10×) were then analyzed (Additional file 6). Similarly to the CCDS regions, the Agilent platforms captured less of the common target regions in total. The regions with poor coverage in the Agilent kits and reasonable coverage in the NimbleGen kits had a higher GC content than the common target regions on average (65.35% in the smaller kits and 66.93% in the updated kits versus mean GC content of 50.71%). These regions also had a higher GC content than the regions that were captured poorly by NimbleGen and reasonably well by Agilent (the GC content in the regions was, respectively, 65.35% versus 59.83% for the smaller kits, and 66.93% versus 62.51% for the updated kits). The regions with poor coverage with NimbleGen and reasonable coverage with Agilent had minutely lower mapability (0.879 versus 0.995 for the smaller kits, and 0.981 versus 0.990 for the updated kits). Both vendors' updated kits performed better in the regions with high GC content or low mapability than the smaller kits.

**Table 3:** GC content of the target regions covered with different sequencing depths

	Mean sequencing coverage of targets			
	
Exome capture method	0×	< 10×	≥ 10×	≥ 20×
Agilent SureSelect	69.00%	64.78%	46.45%	44.52%
Agilent SureSelect 50 Mb	66.39%	65.03%	47.23%	45.01%
NimbleGen SeqCap	69.09%	68.56%	48.54%	47.00%
NimbleGen SeqCap v2.0	68.46%	70.15%	48.89%	47.50%

### SNVs and SNPs

**Figure 4:**
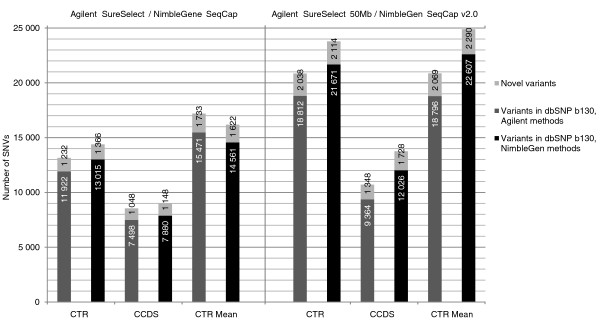
**Number of identified novel and known single nucleotide variants**. SNVs were called with SamTools pileup, and the called variants were filtered based on the allele quality ratio in VCP. Numbers are given for variants with a minimum sequencing depth of 20× in the capture target region (CTR) and CCDS annotated exon regions (CCDS) for the control I sample. Mean numbers for the variants found in the CTRs of the additional samples are also given (CTR Mean). Dark grey bars represent Agilent SureSelect (left panel) and SureSelect 50 Mb (right panel); black bars represent NimbleGen SeqCap (left panel) and SeqCap v2.0 (right panel); light grey bars represent novel SNPs (according to dbSNP b130).

SNVs were called using SAMtools' pileup [13]. In addition to pileup genotype calls, an in-house developed algorithm implemented in the VCP was used to re-call these genotypes. The VCP algorithm takes advantage of allele quality ratios of bases in the variant position (see the 'Computational methods' section). Genome-wide, we found 26,878 ≥ 20× covered SNVs with Agilent SureSelect, 42,799 with Agilent SureSelect 50 Mb, 25,983 with NimbleGen SeqCap and 56,063 with NimbleGen SeqCap v2.0 with approximately 58 million 82-bp high-quality reads in the control I sample. In the additional 25 samples the numbers of found variants were higher for the small exome capture kits than in the control I sample: genome-wide, 42,542, 43,034, 33,893 and 50,881 SNVs with a minimum coverage of 20× were found on average with 59 million reads, respectively. Figure 4 shows the number of novel and known SNVs identified in the CTR and CCDS regions for the control I sample and the mean number of novel and known SNVs in the CTR for the additional samples. The mean allele balances for the heterozygous variants were examined genome-wide and within the CTRs for the control I sample as well as for the additional samples. Interestingly, heterozygous SNVs within the CTRs showed higher allele ratios, indicating a tendency to have more reference bases than variant bases in the variant positions, while the allele balances of the SNVs mapping outside the CTRs were more equal (Table 4). Moreover, allele balances tended to deviate more from the ideal 0.5 towards the reference call with increasing sequencing depth (Additional file 7).

**Table 4:** Mean allele balances of heterozygous SNVs genome-wide and in CTRs

		Control I		Additional samples		
				
Exome capture method	Number of samples	Genome-wide^a^	CTR^b^	Genome-wide^a^	CTR^b^	Student's *t*-test *P*^c^
Agilent SureSelect	3	0.517	0.524	0.511	0.517	0.007
Agilent SureSelect 50 Mb	3	0.515	0.520	0.514	0.519	0.003
NimbleGen SeqCap	20	0.516	0.527	0.514	0.523	7.1 × 10^-15^
NimbleGen SeqCap v2.0	3	0.512	0.518	0.514	0.519	0.013

^a^All called heterozygous SNVs with minimum sequencing coverage of 20×, regardless of target region. ^b^Heterozygous SNVs with minimum sequencing coverage of 20× called within the CTRs. ^c^Student's *t*-test *P*-value for the difference between CTR and all sequenced regions given for the combined sample set of the control I sample and 25 additional samples.

**Figure 5:**
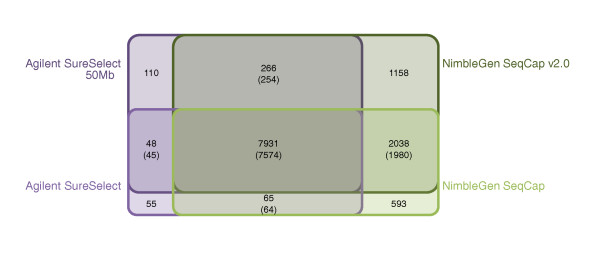
**Sharing of single nucleotide variants between the exome capture kits**. The number of all sequenced variants in the common target region was specified as the combination of all variants found with a minimum coverage of 20× in any of the exome capture kits (altogether, 15,044 variants). Variable positions were then examined for sharing between all kits, both Agilent kits, both NimbleGen kits, Agilent SureSelect kit and NimbleGen SeqCap kit, and Agilent SureSelect 50 Mb kit and NimbleGen SeqCap v2.0 kit. Numbers for the shared variants between the kits in question are given, followed by the number of shared variants with the same genotype calls. The diagram is schematic, as the sharing between Agilent SureSelect and NimbleGen SeqCap v2.0, Agilent SureSelect 50 Mb and NimbleGen SeqCap or any of the combinations of three exome capture kits is not illustrated.

We next estimated the proportion of variation that each capture method was able to capture from a single exome. This was done by calculating the number of SNVs identified by each kit in the part of the target region that was common to all kits in the control I sample. As this region was equally targeted for sequence capture in all exome kits, ideally all variants from the region should have been found with all the kits. Altogether, 15,044 quality filtered SNVs were found in the common target region with a minimum coverage of 20×. Of these SNVs, 8,999 (59.8%) were found with Agilent SureSelect, 9,651 (64.2%) with SureSelect 50 Mb, 11,021 (73.3%) with NimbleGen SeqCap and 13,259 (88.1%) with SeqCap v2.0. Sharing of SNVs between the kits is presented in Figure 5. Of the 15,044 variant positions identified with any method in the common target region, 7,931 were covered with a minimum of 20× coverage by all four methods, and 7,574 (95.5%) of them had the same genotype across all four methods. Most of the remaining 357 SNVs with discrepant genotypes had an allele quality ratio close to either 0.2 or 0.8, positioning them in the 'grey zone' between the clear genotype clusters, thus implying an accidental designation as the wrong genotype class. For the majority of the SNVs (*n *= 281) only one of the capture methods disagreed on the genotype, and the disagreements were randomly distributed among the methods. Agilent SureSelect had 51, SureSelect 50 Mb 87, NimbleGen SeqCap 98 and SeqCap v2.0 45 disagreeing genotypes.

In order to assess the accuracy of the identified variants, we compared the sequenced genotypes with genotypes from an Illumina Human660W-Quad v1 SNP chip for the control I sample. From the SNPs represented on the chip and mapping to a unique position in the reference genome, 11,033 fell inside the Agilent SureSelect CTR, 14,286 inside the SureSelect 50 Mb CTR, 9,961 inside the NimbleGen SeqCap CTR and 12,562 inside the SeqCap v2.0 CTR. Of these SNPs, Agilent SureSelect captured 6,855 (59.7%) with a minimum sequencing coverage of 20×, SureSelect 50 Mb captured 8,495 (59.5%), NimbleGen SeqCap captured 7,436 (74.7%) and SeqCap v2.0 captured 9,961 (79.3%). The correlations of sequenced genotypes and chip genotypes were 99.92%, 99.94%, 99.89% and 99.95%, respectively. The number of concordant and discordant SNPs and genotype correlations for lower sequencing depths are shown in Table 5.

**Table 5:** Genotype correlations with the genome-wide SNP genotyping chip for lower sequencing coverages

	Sequencing depth 1× to 5×			Sequencing depth 6× to 10×			Sequencing depth 11× to 15×		
			
Exome capture method	Number of concordant SNPs	Number of discordant SNPs	Genotype correlation	Number of concordant SNPs	Number of discordant SNPs	Genotype correlation	Number of concordant SNPs	Number of discordant SNPs	Genotype correlation
Agilent SureSelect	779	258	75.12%	802	46	94.58%	647	17	97.44%
Agilent SureSelect 50 Mb	846	243	77.69%	1,127	37	96.82%	1,109	14	98.75%
NimbleGen SeqCap	206	60	77.44%	361	19	95.00%	459	13	97.25%
NimbleGen SeqCap v2.0	110	39	73.83%	338	9	97.41%	486	3	99.39%

**Figure 6:**
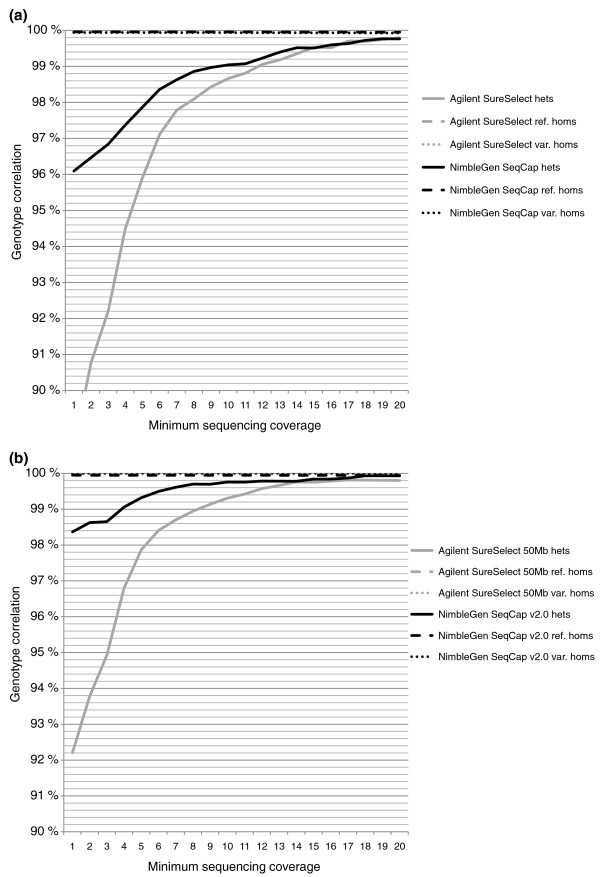
**Correlation of sequenced genotypes to the SNP chip genotypes**. SAMtools' pileup genotype calls recalled with quality ratios in the VCP were compared with the Illumina Human660W-Quad v1 SNP chip genotypes. **(a) **The correlations for Agilent SureSelect- and NimbleGen SeqCap-captured sequenced genotypes. **(b) **The correlations for SureSelect 50 Mb- and SeqCap v2.0-captured sequenced genotypes. Correlations for heterozygous, reference homozygous and variant homozygous SNPs (according to the chip genotype call) are presented on separate lines, though the lines for homozygous variants, laying near 100% correlation, cannot be visualized. The x-axis represents the accumulative minimum coverage of the sequenced SNPs.

We further examined the correlation separately for reference homozygous, variant homozygous and heterozygous SNP calls based on the chip genotype. The cause of most of the discrepancies between the chip and sequenced genotype turned out to be heterozygous chip genotypes that were called homozygous reference bases in the sequencing data, though the number of differing SNPs was too small to make any definite conclusions. Forty-seven of the discordant SNPs were shared between all four exome capture methods with a reasonably deep (≥ 10×) sequencing coverage for SNP calling. Only two of these SNPs had the same VCP genotype call in all four methods, indicating probable genotyping errors on the chip. One SNP was discordant in two methods (Agilent SureSelect and NimbleGen SeqCap), and the rest of the discordant SNPs were discordant in only one method, suggesting incorrect genotype in the sequencing: 12 SNPs in Agilent SureSelect, 26 in SureSelect 50 Mb and 6 in NimbleGen SeqCap. Figure 6 shows the genotype correlation with different minimum sequencing coverages. Additional file 8 presents the correlations between the sequenced genotype calls and chip genotypes with the exact sequencing coverages. Reasons for differences between the methods in the genotype correlation with the lower sequencing depths were examined by determining GC content and mapability for the regions near the discordant SNPs. As expected, GC content was high for the SNPs with low sequencing coverage. Yet there was no difference in the GC content between concordant and discordant SNPs. Additionally, we did not observe any remarkable difference in the GC content of concordant and discordant SNPs between the different capture methods, independent of sequencing coverage (data not shown). Mapabilities for all the regions adjacent to the discordant SNPs were 1.0; thus, they did not explain the differences. Despite the allele balances for the heterozygous variants being closer to the ideal 0.5 outside the CTRs than within the CTRs, there was no notable improvement in the genotype correlation when examining SNPs in the regions with more untargeted base pairs (data not shown).

Correlations between the original SAMtools' pileup [13] genotypes and the chip genotypes, as well as correlations for genotypes called with the Genome Analysis Toolkit (GATK) [18], were also examined and are given in Additional file 9. Recalling of the SNPs with quality ratios in the VCP greatly enhanced the genotype correlation of heterozygous SNPs from that of the original SAMtools' pileup genotype correlation. For the heterozygous SNPs, GATK genotypes correlated with the chip genotypes slightly better than the VCP genotypes with low sequencing coverages (5× to 15×), especially for the smaller versions of the capture kits. However, correlation of the variant homozygous SNPs was less accurate when GATK was used.

### Insertion-deletions

Small indels variations were called with SAMtools pileup for the control I sample. Altogether, 354 insertions and 413 deletions were found in the CTR of Agilent SureSelect, 698 insertions and 751 deletions in the CTR of SureSelect 50 Mb, 365 insertions and 422 deletions in the CTR of NimbleGen SeqCap and 701 insertions and 755 deletions in the CTR of SeqCap v2.0, with the minimum sequencing coverage of 20×. The size of the identified indels varied from 1 to 34 bp. There was practically no difference in the mean size of the indels between the capture methods. Of all 2,596 indel positions identified with any one of the methods, 241 were identified by all four methods, 492 by any three methods and 1,130 by any two methods; 119 were identified only with Agilent SureSelect, 619 only with SureSelect 50 Mb, 149 only with NimbleGen SeqCap and 579 only with SeqCap v2.0. We further attempted to enhance the identification of indels by searching for positions in the aligned sequence data where a sufficient number of overlapping reads had the same start or end position without being PCR duplicates (see the 'Computational methods' section). These positions were named as REAs. We found 40 REAs in the CTR of Agilent SureSelect, 157 in the CTR of SureSelect 50 Mb, 53 in the CTR of NimbleGen SeqCap and 92 in the CTR of SeqCap v2.0. Only four of these REAs were found with all four methods, despite 110 of them being in the common region targeted for capture in all. Agilent's capture methods shared 27 REAs and NimbleGen's methods shared 19 REAs. Of the indels identified with pileup, 30% overlapped with known indels from dbSNP b130 and 43% of the REAs overlapped with a known copy number variation (Database of Genomic Variants, March 2010 freeze). Extensive validation of the found indels is needed for the evaluation of the algorithms.

### Simulation of exome sequencing in monogenic diseases

Finally, we evaluated the exome capture kits' potential in finding a set of disease-causing mutations of monogenic disorders. Using 48 previously published mutation loci of 31 clinically relevant disorders of the Finnish disease heritage (references are given in the Additional file 10) as an example, we examined whether the methods had successfully and reliably captured these genomic positions in the control I sample. With a minimum coverage of 10×, Agilent SureSelect captured 34 of the mutation loci, SureSelect 50 Mb captured 34, NimbleGen SeqCap 39 and SeqCap v2.0 captured 42 of the mutation loci. When the threshold was raised to ≥ 20× coverage, the kits captured 30, 30, 34 and 37 disease-causing mutation loci, respectively. Four loci were missed by all the kits despite the loci being within the CTR of each kit. Of note, no mutant alleles were found in any of the covered loci for the control I sample. Additional file 10 shows the examined diseases, genomic positions of the mutations, mutation types and the sequencing coverage of different exome capture kits on the loci.

## Discussion

Our results show more specific targeting and enrichment characteristics for sequencing libraries captured with the Roche NimbleGen exome capture kits than for libraries captured with the Agilent kits. Although sequences of the libraries prepared using the Agilent kits had less duplicated reads and their aligning to the human reference genome was equal to that of the NimbleGen kits, the latter had more high quality reads and deeply covered base pairs in the regions actually targeted for sequence capture. The alignment results indicate a more widespread distribution of the sequencing reads from Agilent kits within the genome.

High GC content of the target regions correlated with low sequencing coverage in all exome capture methods. The GC content seemed to affect Agilent's long RNA-based probes slightly more than NimbleGen's DNA-based probes, but it did not solely explain the difference in capture success between the methods. Carefully balanced probe design with shorter and more numerous probes in NimbleGen's kits seemed to provide a more uniform coverage throughout the target regions, including the challenging areas.

Evaluation of the allele balances of the identified heterozygous SNVs revealed no major differences between the NimbleGen and Agilent capture methods. However, we observed that the variations outside the CTRs had a more ideal balance, close to 0.5, than the heterozygous variations in the CTRs. This was true for both exome capture method vendors. This suggests that the capture probes, being specific for the reference sequence, favor the reference alleles in the hybridization and capture processes. SNVs identified outside the CTRs are captured because of the overflow of sequencing fragments beyond the targeted regions, and thus are not under the selection of an annealing probe. Furthermore, deviation from 0.5 increased with increasing sequencing depth. Both vendors slightly improved their allele balances in their updated capture kits.

SNP correlation with the Illumina Human660W-Quad v1 SNP chip was not notably different between the exome capture methods. All methods captured the SNPs with a high correlation of more than 99.7% when a minimum sequencing depth of 20× was used. When the allele quality ratios were considered in the SNP calling, over 99% correlation with common SNPs represented on the genotyping chip was already achieved with an approximate minimum sequencing depth of 10×. However, common SNPs on genome-wide association arrays are biased towards easy-to-genotype SNPs, and novel variants likely need a deeper sequencing coverage for an accurate genotype.

The number of captured CCDS exons and transcripts and found SNVs closely followed the success rate of the short read alignment in the region of interest. This was also seen with indel variations and how the methods captured the previously identified mutation loci of the Finnish disease heritage. As all the following sequence analysis steps were dependent on the sequencing depth, deep and uniform sequencing coverage of the CTR is essential for the sequence capture method's performance. This makes the normalization of read counts a crucial step for a systematic comparison. We chose to use comparable amounts of effective reads (that is, high quality, not duplicated reads) in the read alignment. The possible effect the different sample preparation methods had on the need for sequencing read trimming and duplicate removal was potentially minimized with this approach, and allowed us to carry out the comparison chiefly on the kits' target enrichment characteristics.

Teer *et al. *[19] used the number of filtered reads in the normalization of their data in a comparison of Agilent SureSelect custom capture, Roche NimbleGen microarray-based capture and molecular inversion probe capture of custom non-contiguous targets, exons and conserved regions. According to their results, NimbleGen microarray-based capture was the most sensitive method. On the other hand, Kiialainen *et al. *[20] came to a different conclusion in their comparison of Agilent SureSelect custom capture and Roche NimbleGen microarray capture methods targeted at 56 genes, including exons, introns and sequences upstream and downstream of the genes. More sequencing reads from their Agilent captures aligned to the CTR compared to their NimbleGen captures. The regions targeted for capture were rather different in these two comparisons, the region in Teer *et al. *possibly resembling more the whole exome target. This suggests that capture probe design with shorter probes of flexible length might be more easily applied to non-contiguous targets. However, Mamanova *et al. *[21] stated in their review on sequence capture methods that no appreciable differences were noticed between the performances of Agilent SureSelect and NimbleGen SeqCap solution-based methods.

We made some modifications to the protocols provided by the vendors for equalization purposes. It can be hypothesized that these modifications could have altered the balance of target DNA and the capture probes in the hybridization, and by this mechanism the subsequent alignment of short reads into the CTRs. Moreover, Fisher *et al. *[22] showed in their study on automation of the Agilent SureSelect sequence capture procedure that the mapping sensitivity and specificity of the kit can be improved with extensive optimization.

Only one of our samples was captured with all four exome capture methods. Although we observed some sample-specific variation in the 25 samples captured with only one method, the mean values across these additional samples were consistent with the values of the control I sample. The observed differences in the number of duplicated reads, the number of reads mapping to the CTR and the percentage of the CTR covered by at least 20 reads between Agilent SureSelect and NimbleGen SeqCap kits were statistically significant.

## Conclusions

When their limitations are acknowledged, whole exome sequence capture kits are an efficient method to target next-generation sequencing experiments on the best understood regions of the genome. One obvious limitation is that none of the capture kits were able to cover all the exons of the CCDS annotation, although there has been improvement in this in the updated versions of the kits. An additional shortage is the lack of targeting of the 5' and 3' untranslated regions, especially in studies of complex diseases, in which protein coding sequences are not necessarily expected to be altered. We found no major differences in the performance of the kits regarding their ability to capture variations accurately. In our data, libraries captured with NimbleGen kits aligned more accurately to the target regions. NimbleGen Seqcap v2.0 most efficiently covered the exome with a minimum coverage of 20×, when comparable amounts of sequence reads were produced from all four capture libraries.

## Materials and methods

### Samples

The control I sample was an from anonymous blood donor. The DNA was extracted from the peripheral blood using a standard method based on salt precipitation at the Public Health Genomics, National Institute for Health and Welfare, Helsinki, Finland. In addition, we estimated the performance of different exome capture methods by auditing the quality and quantity of exome sequencing data produced for purposes of five on-going research projects employing the herein described core-facility services. Each research project was approved by an Ethics Committee (Ethics Committees of the Helsinki University Central Hospital and Bioethics Committee of the Institute of Oncology, Maria Sklodowska-Curie, Warsaw). All samples were taken in accordance with the Helsinki Declaration, with oral or written consent from the patients or their parents. All samples were processed anonymously, and the samples were prepared and analyzed in our core-facility laboratory using the same protocols. This auditing allowed us to compare the overall performance of different exome capture methods, and to monitor the quality of the sequence data. Two of the additional samples were prepared and captured with the Agilent SureSelect Human All Exon kit, two with the Agilent SureSelect Human All Exon 50 Mb kit, 19 with the NimbleGen SeqCap EZ Exome kit and two with the NimblGen SeqCap EZ Exome v2.0 kit. DNA was extracted from the samples in the respective laboratory responsible for each research project using standard protocols.

### Sample preparation I

For sample preparation I (control I sample, Additional file 11a), two sets of 3 μg of DNA were fragmented with a Covaris S-2 instrument (Covaris, Woburn, MA, USA), purified with QIAquick PCR purification columns (Qiagen, Hilden, Germany) and pooled together. Fragmentation success was verified by running 4 μl of the sample on a FlashGel (Lonza, Allendale, NJ, USA). The rest of the sample was divided, and the end repairing, A-tailing and adapter ligation and the concomitant column purifications were done in parallel for the divided sample with NEBNext DNA Sample Prep Master Mix Set 1 (New England BioLabs, Ipswich, MA, USA) using the concentrations recommended by the manufacturer and the Qiagen purification columns. For the adapter ligation, adapters were formed from primers 5'-GATCGGAAGAGCGGTTCAGCAGGAATGCCGAG-3'and 5'-ACACTCTTTCCCTACACGACGCTCTTCCGATCT-3' (oligonucleotide sequences ^© ^2006-2008 Illumina, Inc., Allendale, NJ, USA, all rights reserved) by mixing 5 nmol of both primers, heating to 96°C for 2 minutes and cooling down to room temperature. Twenty-five pmol of the adapter was used for the ligation reaction. After completion of sample preparation, the samples were first pooled and then split to ascertain a uniform starting product for both sequence capture methods.

For the NimbleGen SeqCap EZ Exome capture (later referred to as NimbleGen SeqCap; Roche NimbleGen, Madison, WI, USA), the adapter-ligated sample was run on a 2% TBE-agarose gel, following which a gel slice containing 200 to 300 bp of DNA was extracted, purified with a QIAquick Gel Extraction column (Qiagen) and analyzed on a Bioanalyzer High Sensitivity DNA chip (Agilent, Santa Clara, CA, USA). Twenty nanograms of the sample was mixed with 25 μl of 2× Phusion HF PCR Master Mix (Finnzymes, Espoo, Finland), 1.2 μl of 20 μM forward and reverse PE PCR primers (5'-AATGATACGGCGACCACCGAGATCTACACTCTTTCCCTACACGACGCTCTTCCGATCT-3' and 5'-CAAGCAGAAGACGGCATACGAGATCGGTCTCGGCATTCCTGCTGAACCGCTCTTCCGATCT-3' (oligonucleotide sequences ^© ^2006-2008 Illumina, Inc., all rights reserved). ddH2O was added to reach the final reaction volume of 50 μl to be used for four parallel reactions in the pre-capture PCR. The cycling conditions were as follows: initial denaturation at 98°C for 2 minutes; 8 cycles of 98°C for 20 seconds, 65°C for 30 seconds and 72°C for 30 seconds; final extension at 72°C for 5 minutes, and cooling down to 10°C until further use. The PCR products were pooled together, purified with a QIAquick PCR purification column and analyzed on a Bioanalyzer DNA1000 chip (Agilent). One microgram of the product was prepared for hybridization with the capture oligomeres; the hybridization was carried out at 47°C for 70 hours and the product was captured using Streptavidin M-270 Dynabeads (Invitrogen, Carlsbad, CA, USA) according to the NimbleGen SeqCap protocol.

For the Agilent SureSelect Human All Exon capture (later referred to as Agilent SureSelect), the adapter-ligated sample was purified using Agencourt AMPure XP beads (Beckman Coulter, Brea, CA, USA) and analyzed on a Bioanalyzer High Sensitivity DNA chip. Twenty nanograms of the sample was used for pre-capture PCR in four parallel reactions in the same conditions as for the NimbleGen SeqCap. The PCR products were pooled together, purified with a QIAquick PCR purification column and analyzed on a Bioanalyzer DNA1000 chip. Five-hundred nanograms of the sample was prepared for the hybridization with the capture baits, and the sample was hybridized for 24 hours at 65°C, captured with the Streptavidin M-280 Dynabeads and purified using a Qiagen MinElute column according to the manufacturer's protocol.

After hybridization and capturing the DNA with streptavidin beads, the captured yield was measured using quantitative PCR. A standard curve was created using a previously prepared Illumina GAIIx sequencing sample with known concentrations of DNA ranging from 0.3 pg/μl to 21.5 pg/μl. One microliter of both capture sample and each control sample solutions were used in triplicate PCR reactions, performed with a DyNAmo HS SYBRGreen qPCR kit (Finnzymes) and PCR primers specific for the PE sequencing primer tails (5'-ATACGGCGACCACCGAGAT-3' and 5'-AGCAGAAGACGGCATACGAG-3'), and run on a LightCycler^® ^480 Real-Time PCR system (Roche NimbleGen). The original DNA concentrations of the capture samples were calculated from the standard curve; 246 pg of DNA was captured with the Agilent SureSelect baits and 59 pg with the NimbleGen SeqCap probes.

After finding out the DNA concentrations of the captured samples, the PCR conditions were optimized for the post-capture PCR-reactions. The most comparable libraries, defined as uniform library sizes and equivalent yields, were obtained by using 5 pg of the captured sample and 14 cycles of PCR for the NimbleGen SeqCap and 10 pg of the captured sample and 16 cycles of PCR for the Agilent SureSelect. Stratagene Herculase II enzyme (Agilent) was used for both PCRs. For the NimbleGen SeqCap, primers 5'-AATGATACGGCGACCACCGAGA-3' and 5'-CAAGCAGAAGACGGCATACGAG-3' were used at a concentration of 100 pmol. For the Agilent SureSelect, a primer mix from the SureSelect kit was used as recommended by the manufacturer. Six parallel reactions were done for both of the exome capture methods, the PCR products were purified according to the exome kit protocols (AMPure SPRI-beads for the Agilent SureSelect sample and QIAquick PCR purification columns for the NimbleGen SeqCap sample), following which the purified PCR products were pooled and analyzed on a Bioanalyzer High Sensitivity DNA chip. The samples were diluted to a concentration of 10 nM, and equal amounts of the libraries were run on an Illumina GAIIx sequencing instrument according to the manufacturer's protocol using PE sequencing.

### Sample preparation II: exome kit updates

For sample preparation II (Additional file 11b), we introduced 6 μg of control I DNA for fragmentation in two batches. After fragmentation, the batches were pooled in order to obtain a highly uniform product for both updated capture kits, as well as for the end repair, adapter ligation and PCR steps, which were conducted as described above. After each step the samples were purified with Agencourt AMPure XP beads. One microgramg of the sample library was hybridized with Roche NimbleGen SeqCap EZ v2.0 probes and 500 ng of the sample library with Agilent SureSelect Human All Exon 50 Mb baits. The hybridizations and captures were performed according to the manufacturers' updated protocols. Quantitative PCR was performed as described in the 'Sample preparation I' section. DNA (525 pg) was captured with Agilent 50 Mb baits and 210 pg with NimbleGen v2.0 baits. The post-capture steps were performed as in the 'Sample preparation I' section.

### Sequencing

Agilent SureSelect and NimbleGen SeqCap sequencing libraries from sample preparation I were sequenced on two lanes each; one lane with a read length of 60 bp and another with 82 bp. As the recommended sequencing length for all of the exome capture kits was 75 bp at the minimum, only the data from the second sequencing lanes of Agilent SureSelect and NimbleGen SeqCap sequencing libraries were used in the analyses proceeding from the alignment of individual lanes. Sequencing libraries captured with the Agilent SureSelect 50 Mb and NimbleGen SeqCap v2.0 kits during sample preparation II were first sequenced on a single lane each. As this resulted in incomparable read amounts (only 42 million reads were produced by the Agilent SureSelect 50 Mb, whereas 85 million reads were obtained from the NimbleGen SeqCap v2.0), another sequencing lane was produced for the SureSelect 50 Mb. Data from the two Agilent SureSelect 50 Mb kit sequencing lanes were combined, and the sequencing reads were randomly down-sampled to meet comparable read amounts after the trimming of B blocks from the read ends and the removal of PCR duplicates. Both lanes for SureSelect 50 Mb were produced with a sequencing length of 82 bp. The NimbleGen SeqCap v2.0 capture library was sequenced with a read length of 100 bp and the reads were trimmed to 82 bp prior to any other action. All raw sequence data can be obtained from the Sequence Read Archive (SRA) with study accession number [SRA:ERP000788] [23].

### SNP-chip

In order to evaluate the exome capture methods' ability to genotype common SNPs, the control I sample was genotyped on an Illumina Human660W-Quad v1 SNP chip in the Technology Centre of the Institute for Molecular Medicine, Finland, according to the manufacturer's protocol. Genotypes were called using GenomeStudio v2009.2. SNPs with < 95% genotyping success rate were excluded from further analyses. To enable comparison of the chip and sequenced genotypes, all flanking sequences of the chip SNPs (provided by the manufacturer) were first aligned with Exonerate software [24] against the human genome build hg19 (GRCh37). Genotypes of the SNPs with a flanking sequence mapping to the minus strand were then reversed to their reverse complements. SNPs with multiple blasting results or no results at all (*n *= 10 047) were removed from further analyses.

### Computational methods

Human genome build hg19 (GRCh37) Primary Assembly (not including the unplaced scaffolds) was used as the reference sequence throughout the analyses. Both Agilent and NimbleGen have used exon annotations from the CCDS and miRNA annotations from the miRBase based on human genome build hg18 as the basis for their capture designs in the smaller kits. In the probe designs for the larger kits, Agilent has used the CCDS (March 2009), GENCODE, RefSeq, Rfam and miRBase v.13 annotations based on human genome hg19, whereas the NimbleGen SeqCap v2.0 design relies on the CCDS (September 2009), RefSeq (UCSC, January 2010), and miRBase (v.14, September 2009) annotations, as well as on additional genes from customer inputs. The updated kits included capture probes for unplaced chromosomal positions as well (namely, 378 probe regions in Agilent SureSelect 50 Mb and 99 in NimbleGen SeqCap v2.0), but these regions were removed from our further analyses. CTRs were defined for all of the capture kits as the companies' given probe positions. These needed to be lifted over from the given hg18 build positions to the recent hg19 positions for the smaller kits, whereas the updated kits' designs had already been made using the hg19 build. In some of our statistics (see Results), we included the flanking 100 bp near all the given probe positions into the CTRs (CTR + flank). Exon annotations from the CCDS project build v59 (EnsEMBL) were used [10]. A common target region for the capture methods was defined as the probe regions that were included in all of the probe designs.

For the probe design comparisons (Figure 1; Additional file 1), the exon regions of interest were defined by combining CCDS and UCSC known exon [11] annotated regions as well as all the kits' capture target regions into a single query. Overlapping genomic regions were merged as single positions in the query. For any given kit, an exon region was considered to be included in the kit if its capture probe positions overlapped with the combined query for one base pair or more. The numbers of included exon regions are given in the figures.

All sequence data were analyzed using an in-house developed SAMtools-based bioinformatics pipeline for quality control, short read alignment, variant identification and annotation (VCP; Figure 2). Image analyses and base calling of the raw sequencing data were first performed on the Illumina RTA v1.6.32.0 sequence analysis pipeline. In the VCP, the sequences were then trimmed of any possible B block in the quality scores from the end of the read. After this, if any pair had a read shorter than 36 bp, the pair was removed. The quality scores were converted to Sanger Phred scores using Emboss (version 6.3.1) [25] and aligned using BWA (version 0.5.8 c) [12] against human genome build hg19. The genome was downloaded from EnsEMBL (version 59). After alignment, potential PCR duplicates were removed with Picard MarkDuplicates (version 1.32).

SNVs were called with SAMtools' pileup (version 0.1.8) [13]. The pileup results were first filtered by requiring the variant allele quality to be 20 or more and then with the SAMtools' VarFilter. We calculated quality ratios for the variants as a ratio of A/(A + B), where A and B were defined as follows: if there were call bases of both the reference base and variant base in the variant position, A was the sum of allele qualities of the reference call bases and B was the sum of allele qualities of the variant call bases; if there were two different variant call bases and no reference call bases, the variant call base with a higher allele quality sum was the A and the other call base was the B; if all the call bases in the variant position were variant calls of the same base, the quality ratio was defined to be 0. In variant positions with call bases of more than two alleles the ratio was defined to be -1, and they were filtered from subsequent analyses. Finally, single nucleotide variants called by pileup were filtered in the VCP according to the described quality ratio: any variant call with a quality ratio of more than 0.8 was considered as a reference call and was filtered out. In addition, we included our own base calls for the called variants based on the quality ratio. Any call with a quality ratio between 0.2 and 0.8 was considered to be heterozygous and calls below 0.2 to be homozygous variant calls.

For the control I sample, GATK base quality score recalibration and genotype calling was done with recommended parameter settings for whole exome sequencing [18]. Known variants for quality score recalibration were from the 1000 Genomes Project (phase 1 consensus SNPs, May 2011 data release).

In addition to SNVs, small indels were called for the control I sample using SAMtools' pileup as well. The results were filtered by requiring the quality to be 50 or more and then with the SAMtools' VarFilter. No other alleles than the indel or reference allele calls were allowed for the indel variant positions.

We hypothesized that indel, inversion or translocation break points could be identified from the aligned sequence data by examining genomic positions, where a sufficient number of overlapping reads had the same start or end position without being PCR duplicates. Such positions could be caused by soft-clipping of reads done by BWA: if only the start of a read aligned to the reference sequence, but the rest of the read did not align adjacently to it, BWA aligned only the start of the read and reported a soft-clip from the un-aligned part. Another possible cause for these positions was B blocks in the quality scores, starting from the same position for the overlapping reads, and subsequent B block trimming. These positions were named as REAs. REAs were searched for in the control I sample from the aligned read file. At least five reads, all of them either starting or ending in the same position, and a minimum contribution of 30% to the total coverage in the position, were required for a REA to be reported. Associated soft-clipped sequences were reported together with REAs.

GC content was defined for the CTRs and the common target region as a mean percentage of G and C bases in the targets, calculated from human genome build hg19 (GRCh37) based FASTA formatted target files with the Emboss geecee script [25]. For the SNP analyses, GC content was defined as the percentage of G and C bases in the distinct target (for example, a single exon) adjacent to the SNP. Mapabilities were retrieved from the UCSC Table Browser using track: mapability, CRG Align 75 (wgEncodeCrgMapabilityAlign75mer). In this track, a mapability of 1.0 means one match in the genome for k-mer sequences of 75 bp, 0.5 means two matches in the genome and so on. Mean mapability was calculated for each distinct target region. Similarly for the SNP analyses, mapability for a SNP was defined as mean mapability in the region adjacent to the SNP.

Student's *t*-test was used to test for statistical significance in the differences between the sequence alignment results and between the SNV allele balances. *T*-distribution and equal variance were assumed for the results, thought it should be noted that with a small number of samples the results should be interpreted with caution. Uncorrected two-tailed *P*-values are given in the text.

## Abbreviations

bp: base pair; BWA: Burrows-Wheeler Aligner; CCDS: Consensus Coding Sequence; CTR: capture target region; GATK: Genome Analysis Toolkit; indel: insertion-deletion; miRNA: microRNA; REA: read end anomaly; SNP: single nucleotide polymorphism; SNV: single nucleotide variant; VCP: Variant Calling Pipeline.

## Competing interests

The authors declare that they have no competing interests.

## Authors' contributions

AMS participated in the study design, sample preparation and the development of the VCP, carried out the statistical analyses and data interpretations and drafted the manuscript. PE participated in the study design, VCP development, statistical analyses and data interpretations. HA carried out the raw sequence analyses, developed the VCP and participated in drafting the manuscript. ML and SH carried out the sample preparations. SE participated in the development of the VCP and drafting the manuscript. TM participated in the SNP chip analysis and helped in the computational methods. HT, PS and CH provided data for the additional samples. HJ, TR and AS provided data for the additional samples and critically reviewed the manuscript. JS designed and supervised the entire study and participated in drafting and editing the manuscript. All authors read and approved the final manuscript.

## Supplementary Material

Additional file 1 **Comparison of the probe designs of the exome capture kits against the CCDS exon annotation, UCSC exon annotation and each other**. **(a) **Numbers of CCDS exon regions, common target regions outside CCDS annotations and the regions covered individually by the Agilent SureSelect and Agilent SureSelect 50 Mb kits. SureSelect has one single region outside the SureSelect 50 Mb design. **(b) **The same as (a) for the NimbleGen SeqCap and NimbleGen SeqCap v2.0 kits. **(c-f) **The same as Figures 1a and 1b and Additional files 1a and 1b, respectively, but the exon annotation from UCSC is given instead of the CCDS annotation. Regions of interest are defined as merged genomic positions, regardless of their strandedness, which overlap with the kit in question. Sizes of the spheres are proportional to the number of targeted regions in the kit. The total number of targeted regions is given under the name flag of each sphere. Click here for file

Additional file 2 **Capture details of the CCDS exons for Agilent SureSelect in the control I sample**. Tabular file listing CCDS annotated exons, their targeting in the Agilent SureSelecet kit and mean sequencing coverage for the control I sample. Click here for file

Additional file 3 **Capture details of the CCDS exons for Agilent SureSelect 50 Mb in the control I sample**. Tabular file listing CCDS annotated exons, their targeting in the Agilent SureSelecet 50 Mb kit and mean sequencing coverage for the control I sample. Click here for file

Additional file 4 **Capture details of the CCDS exons for NimbleGen SeqCap in the control I sample**. Tabular file listing CCDS annotated exons, their targeting in the NimbleGen SeqCap kit and mean sequencing coverage for the control I sample. Click here for file

Additional file 5 **Capture details of the CCDS exons for NimbleGen SeqCap v2.0 in the control I sample**. Tabular file listing CCDS annotated exons, their targeting in the NimbleGen SeqCap v2.0 kit and mean sequencing coverage for the control I sample. Click here for file

Additional file 6 **Comparison of poorly captured targets between the exome capture kits**. Table presenting comparisons between the regions of the common target with poor capture success in one kit (mean sequencing coverage 0×) and reasonable capture success in another kit (mean sequencing coverage ≥ 10×). Click here for file

Additional file 7 **Mean allele balances for heterozygous single nucleotide variants**. **(a-e) **Allele balances are given for the heterozygous SNVs in the whole genome (a), in each exome capture method's own CTR (b), in each exome capture method's own CTR and flanking the 100 bp (c), in the CCDS annotated exon regions (d) and the common regions targeted for capture in all the methods (e) for different minimum sequencing coverages. In (a, b), allele balances are given for the control I sample (bars without outline) and for the mean values from the 26 additional exome samples (bars with thick outline). The ideal allele balance of 0.5 is indicated with a red line. Regions including mostly non-targeted base pairs, as in the whole genome and CTR + flanking regions, had a mean allele balance closer to 0.5 than the regions with only targeted base pairs. Additionally, allele balance was shifted away from the 0.5 with increasing minimum sequencing depth. Click here for file

Additional file 8 **Correlation of VCP genotype calls from Agilent SureSelect- and NimbleGen SeqCap-captured (a) and SureSelect 50 Mb- and SeqCap v2.0-captured (b) sequenced genotypes to the Illumina Human660W-Quad v1 SNP chip genotypes with exact sequencing coverages**. Correlations for heterozygous, reference homozygous and variant homozygous SNPs (according to the chip genotype call) are presented in separate graphs, though graphs lying near 100% correlation cannot be visualized. The x-axis represents the exact coverage of the sequenced SNPs. Click here for file

Additional file 9 **Correlation of SAMtools' pileup genotype calls (a, b) and GATK genotype calls (c, d) from Agilent SureSelect- and NimbleGen SeqCap-captured and SureSelect 50 Mb- and SeqCap v2.0-captured sequenced genotypes to the Illumina Human660W-Quad v1 SNP chip genotypes**. The SAMtools' pileup genotype calls correlated worse with the chip genotypes than the genotypes accommodated with the quality ratios. Correlations for heterozygous, reference homozygous and variant homozygous SNPs (according to the chip genotype call) are presented in separate graphs, though graphs lying near 100% correlation cannot be visualized. The x-axis represents the accumulative coverage of the sequenced SNPs. Click here for file

Additional file 10 **Examined disorders of the Finnish disease heritage, their mutation loci and the sequencing coverage of control sample I on the loci**. Click here for file

Additional file 11 **Sample preparation workflows for sample preparation I (a) and sample preparation II (b)**. **(a) **Orange boxes represent the protocol provided by Agilent for the SureSelect Human All Exon capture kit, and green boxes the protocol for the SeqCap EZ Exome capture kit by NimbleGen. Protocol simplifications and equalizations were made in the sample preparation, and are represented as blue boxes and arrows. **(b) **Similarly for sample preparations II, orange boxes refer to Agilent SureSelect 50 Mb and green boxes to NimbleGen SeqCap v2.0. Not all the steps of the provided protocols are represented. Click here for file
