# Supplementary figures and images for: Comparison of solution-based exome capture methods for next generation sequencing
Source: Genome Biol. 2011 Sep 28;12(9):R94. doi: 10.1186/gb-2011-12-9-r94 (PMC3308057; doi:10.1186/gb-2011-12-9-r94)

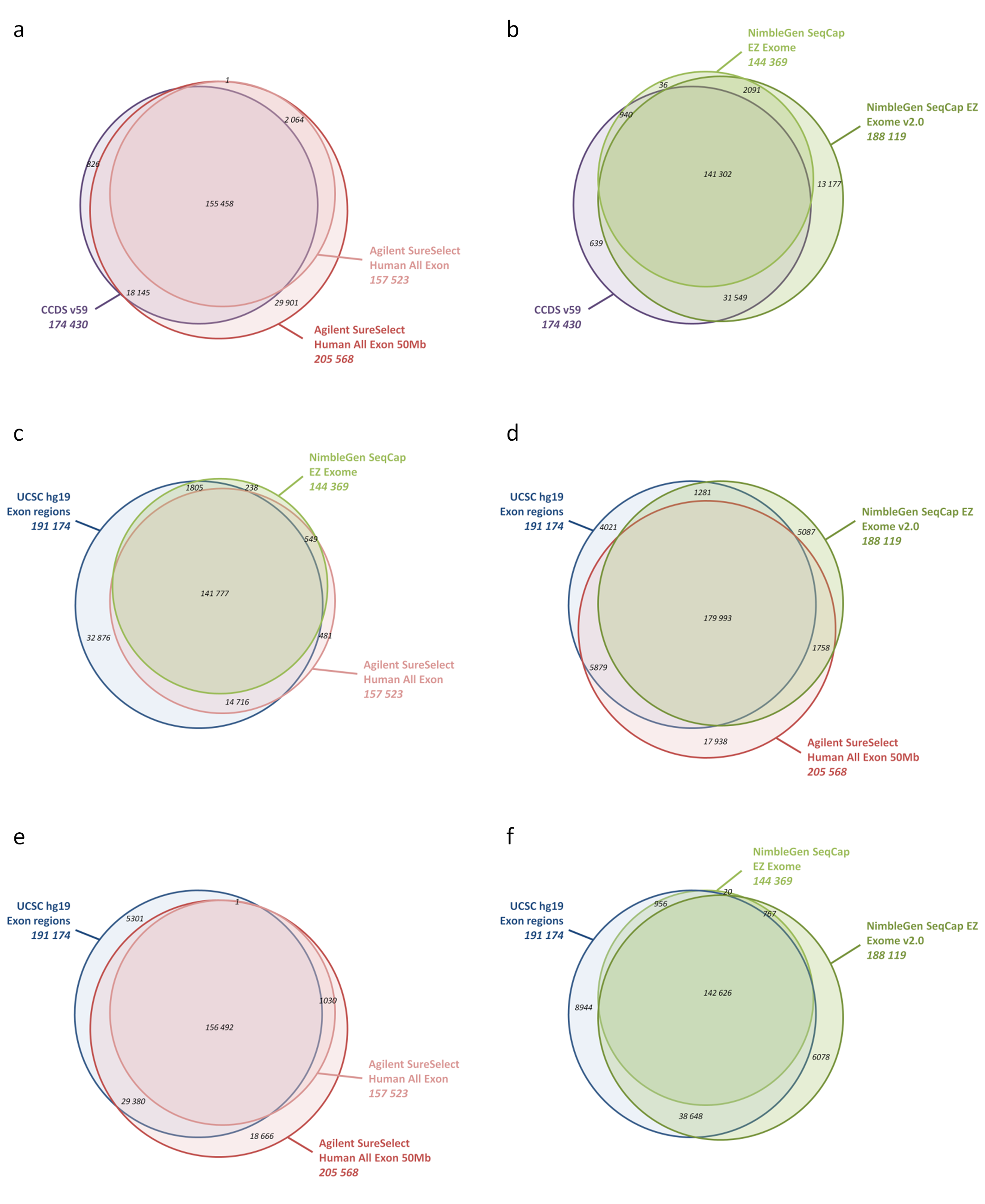

Supplement: Additional file 1 — Comparison of the probe designs of the exome capture kits against the CCDS exon annotation, UCSC exon annotation and each other. (a) Numbers of CCDS exon regions, common target regions outside CCDS annotations and the regions covered individually by the Agilent SureSelect and Agilent SureSelect 50 Mb kits. SureSelect has one single region outside the SureSelect 50 Mb design. (b) The same as (a) for the NimbleGen SeqCap and NimbleGen SeqCap v2.0 kits. (c-f) The same as Figures 1a and 1b and Additional files 1a and 1b, respectively, but the exon annotation from UCSC is given instead of the CCDS annotation. Regions of interest are defined as merged genomic positions, regardless of their strandedness, which overlap with the kit in question. Sizes of the spheres are proportional to the number of targeted regions in the kit. The total number of targeted regions is given under the name flag of each sphere. [file gb-2011-12-9-r94-S1.TIFF]

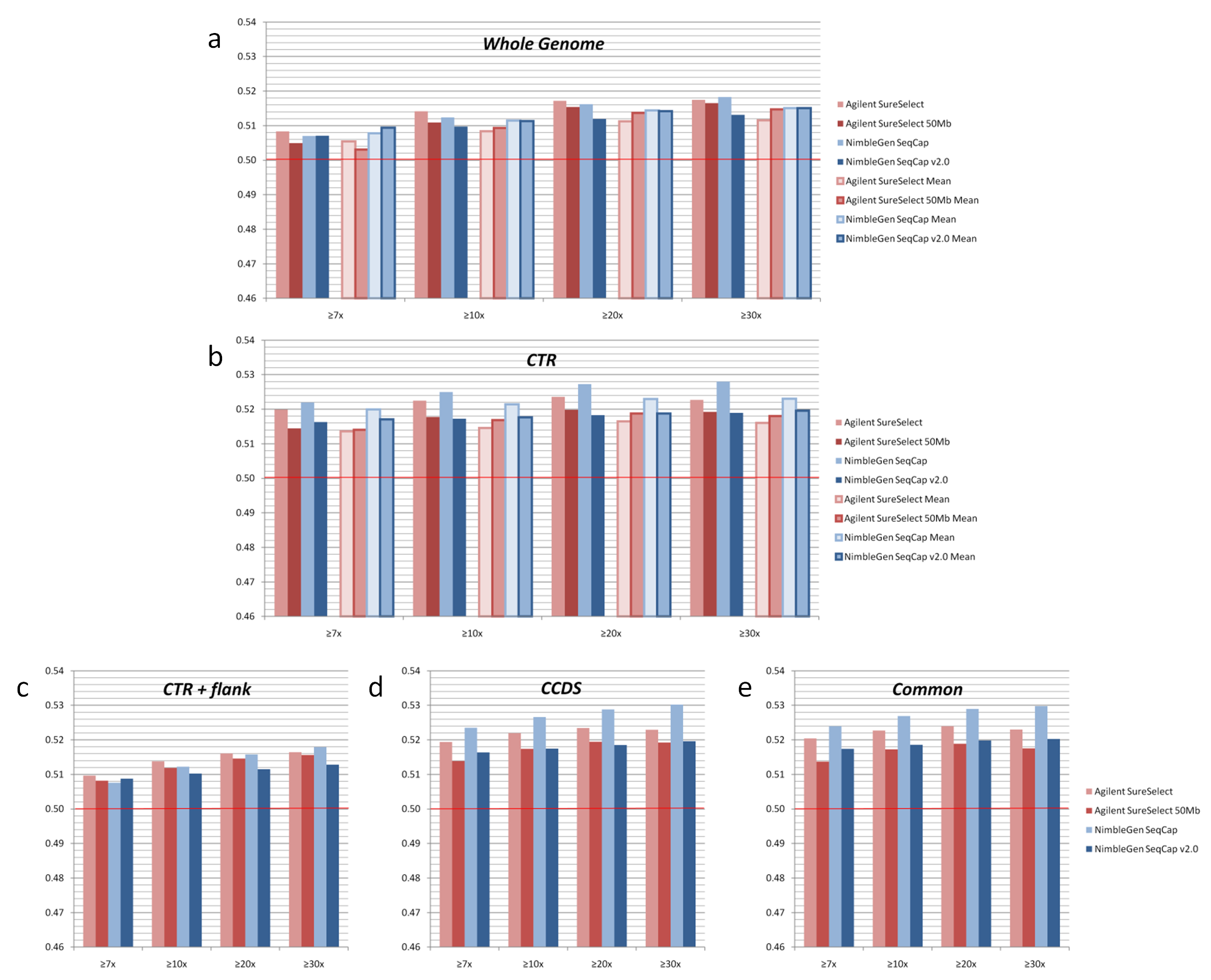

Supplement: Additional file 7 — Mean allele balances for heterozygous single nucleotide variants. (a-e) Allele balances are given for the heterozygous SNVs in the whole genome (a), in each exome capture method's own CTR (b), in each exome capture method's own CTR and flanking the 100 bp (c), in the CCDS annotated exon regions (d) and the common regions targeted for capture in all the methods (e) for different minimum sequencing coverages. In (a, b), allele balances are given for the control I sample (bars without outline) and for the mean values from the 26 additional exome samples (bars with thick outline). The ideal allele balance of 0.5 is indicated with a red line. Regions including mostly non-targeted base pairs, as in the whole genome and CTR + flanking regions, had a mean allele balance closer to 0.5 than the regions with only targeted base pairs. Additionally, allele balance was shifted away from the 0.5 with increasing minimum sequencing depth. [file gb-2011-12-9-r94-S7.TIFF]

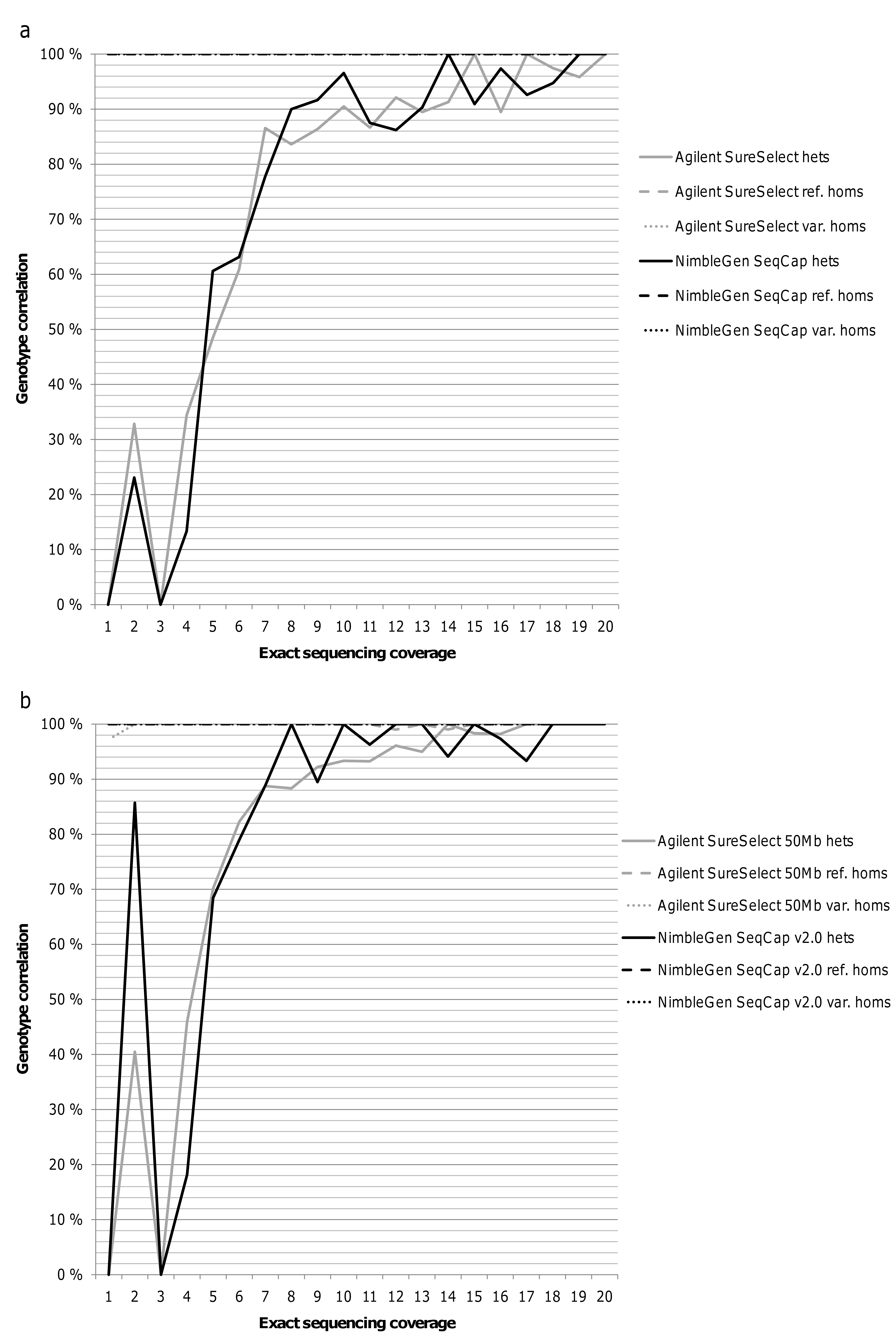

Supplement: Additional file 8 — Correlation of VCP genotype calls from Agilent SureSelect- and NimbleGen SeqCap-captured (a) and SureSelect 50 Mb- and SeqCap v2.0-captured (b) sequenced genotypes to the Illumina Human660W-Quad v1 SNP chip genotypes with exact sequencing coverages. Correlations for heterozygous, reference homozygous and variant homozygous SNPs (according to the chip genotype call) are presented in separate graphs, though graphs lying near 100% correlation cannot be visualized. The x-axis represents the exact coverage of the sequenced SNPs. [file gb-2011-12-9-r94-S8.TIFF]

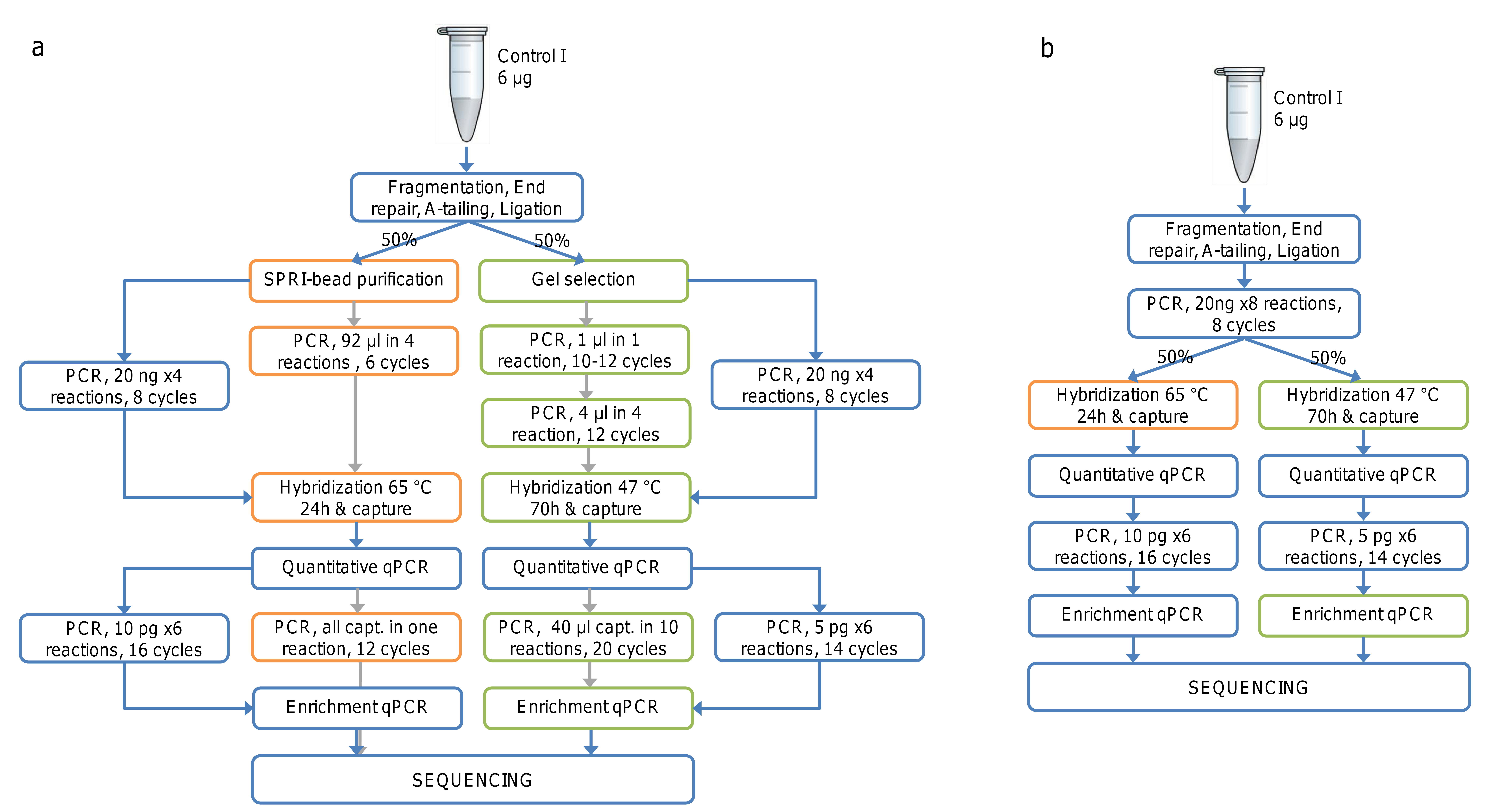

Supplement: Additional file 11 — Sample preparation workflows for sample preparation I (a) and sample preparation II (b). (a) Orange boxes represent the protocol provided by Agilent for the SureSelect Human All Exon capture kit, and green boxes the protocol for the SeqCap EZ Exome capture kit by NimbleGen. Protocol simplifications and equalizations were made in the sample preparation, and are represented as blue boxes and arrows. (b) Similarly for sample preparations II, orange boxes refer to Agilent SureSelect 50 Mb and green boxes to NimbleGen SeqCap v2.0. Not all the steps of the provided protocols are represented. [file gb-2011-12-9-r94-S11.TIFF]
