# Supplementary material for: Comparison of solution-based exome capture methods for next generation sequencing
Source: Genome Biol. 2011 Sep 28;12(9):R94. doi: 10.1186/gb-2011-12-9-r94 (PMC3308057; doi:10.1186/gb-2011-12-9-r94)
Supplement: Additional file 6 — Comparison of poorly captured targets between the exome capture kits. Table presenting comparisons between the regions of the common target with poor capture success in one kit (mean sequencing coverage 0×) and reasonable capture success in another kit (mean sequencing coverage ≥ 10×). [file gb-2011-12-9-r94-S6.PDF]

Additional File 6 - Comparison of poorly captured targets between the exome capture kits

Comparison	Common targets 0x in	and same targets ≥10x in	N. of targets	GC-content	Mapability
Between methods	Agilent SureSelect	NimbleGen SeqCap	2397	65.35%	0.995
	Agilent SureSelect 50Mb	NimbleGen SeqCap v2.0	1082	66.93%	0.990
	NimbleGen SeqCap	Agilent SureSelect	200	59.83%	0.879
	NimbleGen SeqCap v2.0	Agilent SureSelect 50Mb	100	62.51%	0.981
Between versions	Agilent SureSelect	Agilent SureSelect 50Mb	920	64.27%	0.984
	NimbleGen SeqCap	NimbleGen SeqCap v2.0	474	66.11%	0.925
	Agilent SureSelect 50Mb	Agilent SureSelect	126	60.93%	0.988
	NimbleGen SeqCap v2.0	NimbleGen SeqCap	130	65.77%	0.990

Mean mapability for all common targets is 0.980 and mean GC-content is 50.71%. Observations are based on one analysed sample only.
