# Supplementary material for: Comparison of solution-based exome capture methods for next generation sequencing
Source: Genome Biol. 2011 Sep 28;12(9):R94. doi: 10.1186/gb-2011-12-9-r94 (PMC3308057; doi:10.1186/gb-2011-12-9-r94)
Supplement: Additional file 10 — Examined disorders of the Finnish disease heritage, their mutation loci and the sequencing coverage of control sample I on the loci. [file gb-2011-12-9-r94-S10.PDF]

Additional file 10. Examined disorders of the Finnish disease heritage, their mutation loci and the sequencing coverage of sample Control I on the loci

Disorder	Gene	Position	Mutation type	Agilent SureSelect	Agilent SureSelect 50Mb	NimbleGen SeqCap	NimbleGen SeqCap v2.0	Reference
INCL	PPT1	chr1:40557071	c.364A>T	18	8	131	62	Vesa J, *et al.* Nature. 1995 Aug 17;376(6541):584-7
Muscle-eye-brain disease	POMGNT1	chr1:46657769	c.1539+1G>A	13	8	33	14	Diesen C, *et al.* J Med Genet. 2004 Oct;41(10):e115
MCAD deficiency	ACADM	chr1:76226846	c.985A>G	91	59	99	49	Matsubara Y, *et al.* Biochem Biophys Res Commun. 1990 Aug 31;171(1):498-505
LCHAD deficiency	HDAHA	chr2:26418054	c.1528G>C	67	38	49	34	IJlst L, *et al.* Biochim Biophys Acta. 1994 Dec 8;1215(3):347-50
FSH resistant ovaries	FSHR	chr2:49210264	c.566C>T	61	24	292	133	Aittomäki K, *et al.* Cell. 1995 Sep 22;82(6):959-68
Congenital lactase deficiency	LCT	chr2:136564701	c.4170T>A	38	22	66	44[a]	Kuokkanen M, *et al.* Am J Hum Genet. 2006 Feb;78(2):339-44
GRACILE	BCS1L	chr2:219525942	c.232A>G	12	38	15	41	Visapää I, *et al.* Am J Hum Genet. 2002 Oct;71(4):863-76
Usher syndrome, type III	CLRN1	chr3:150645894	c.528T>G	48	81	130	98	Joensuu T, *et al.* Am J Hum Genet. 2001 Oct;69(4):673-84
Usher syndrome, type III	CLRN1	chr3:150659443	c.359T>A	74	29	79	96	Joensuu T, *et al.* Am J Hum Genet. 2001 Oct;69(4):673-84
Meckel syndrome, type 6	CC2D2A	chr4:15538697	c.1762C>T	1[a]	40	0[a]	51	Tallila J, *et al*. Am J Hum Genet. 2008 Jun;82(6):1361-7
Aspartylglucosaminuria	AGA	chr4:178359918	c.488G>C	69	24	115	46	Ikonen E, *et al.* EMBO J. 1991 Jan;10(1):51-8
Aspartylglucosaminuria	AGA	chr4:178361510	c.199_200delGA	79	61	70	65	Isoniemi A, *et al.* Hum Mutat. 1995;5(4):318-26
Diastrophic dysplasia	SLC26A2	chr5:149357190	c.-26+2T>C	63[a]	29[a]	13[a]	27[a]	Hästbacka J, *et al.* Eur J Hum Genet. 1999 Sep;7(6):664-70
Diastrophic dysplasia	SLC26A2	chr5:149359991	c.837C>T	98	52	76	125	Hästbacka J, *et al.* Eur J Hum Genet. 1999 Sep;7(6):664-70
ARPKD	PKHD1	chr6:51524512	c.10412T>G	131	70	78	36	Bergmann C, *et al.* J Am Soc Nephrol. 2003 Jan;14(1):76-89
ARPKD	PKHD1	chr6:51923148	c.1486C>T	22	31	20	92	Bergmann C, *et al.* J Am Soc Nephrol. 2003 Jan;14(1):76-89
ARPKD	PKHD1	chr6:51947999	c.107C>T_	98	28	148	67	Bergmann C, *et al.* J Am Soc Nephrol. 2003 Jan;14(1):76-89
Salla disease	SLC17A5	chr6:74325141	c.1007_1008delTA	44	51	231	73	Aula N, *et al.* Am J Hum Genet. 2000 Oct;67(4):832-40
Salla disease	SLC17A5	chr6:74354306	c.115C>T	64	62	171	63	Verheijen FW, *et al.* Nat Genet. 1999 Dec;23(4):462-5
Argininosuccinic aciduria	ASL	chr7:65557553	c.1153C>T	2	12	6	8	Kleijer WJ, *et al.* J Inherit Metab Dis. 2002 Sep;25(5):399-410
Congenital chloride diarrhea	SLC26A3	chr7:107427293	c.951_953delGGT	43	39	86	96	Höglund P, *et al.* Nat Genet. 1996 Nov;14(3):316-9
Long QT syndrome, type 2	KCNH2	chr7:150648827	c.1655T>C	6	5	9	16	Piippo K, *et al.* J Am Coll Cardiol. 2000 Jun;35(7):1919-25
Long QT syndrome, type 2	KCNH2	chr7:150655537	c.526C>T	0	1	2	0	Laitinen P, *et al.* Hum Mutat. 2000 Jun;15(6):580-1
Northern epilepsy	CLN8	chr8:1719290	c.70C>G	28	21	33	58	Ranta S, *et al.* Nat Genet. 1999 Oct;23(2):233-6
Cohen syndrome	VPS13B	chr8:100454764	c.3348_3349delCT	101	46	145	135	Kolehmainen J, *et al.* Am J Hum Genet. 2003 Jun;72(6):1359-69
Finnish type amyloidosis	GSN	chr9:124073097	c.640G>A	15	7	63	52	Maury CP, *et al.* FEBS Lett. 1990 Jan 15;260(1):85-7
IOSCA	C10ORF2	chr10:102750231	c.1523A>G	29	52	168	39	Nikali K, *et al.* Hum Mol Genet. 2005 Oct 15;14(20):2981-90
Long QT syndrome, type 1	KCNQ1	chr11:2608798	c.1129-2A>G	5	11	33	26	Fodstad H, *et al.* Ann Med. 2004;36 Suppl 1:53-63
Long QT syndrome, type 1	KCNQ1	chr11:2799239	c.1766G>A	20	5	35	18	Piippo K, *et al.* J Am Coll Cardiol. 2001 Feb;37(2):562-8
Hydrolethalus syndrome	HYLS1	chr11:125769895	c.632A>G	66	35	45	52	Mee L, *et al.* Hum Mol Genet. 2005 Jun 1;14(11):1475-88
vLINCL	CLN5	chr13:77566311	c.225G>A	0	1	1	1	Savukoski M, *et al.* Nat Genet. 1998 Jul;19(3):286-8
vLINCL	CLN5	chr13:77575054	c.1175_1176delAT	87	55	93	13[a]	Savukoski M, *et al.* Nat Genet. 1998 Jul;19(3):286-8
JNCL	CLN3	chr16:28491805-28494805	3 kb deletion[b]	8.4	16.4	25.3	58.0	The International Batten Disease Consortium. Cell. 1995 Sep 22;82(6):949-57.
JNCL	CLN3	chr16:28497245-28498245	1.02 kb deletion[c]	2.5	4.0	10.4	19.0	The International Batten Disease Consortium. Cell. 1995 Sep 22;82(6):949-57.
Meckel syndrome, type 1	MKS1	chr17:56283914-56283943	c.1408-7_35del[d]	2.8[e]	4.5[e]	0.9[a]	75.4	Kyttälä M, *et al.* Nat Genet. 2006 Feb;38(2):155-7
Mulibrey nanism	TRIM37	chr17:57157240	c.493-2A>G	106	59	209	144	Avela K, *et al.* Nat Genet. 2000 Jul;25(3):298-301
Congenital nephrosis	NPHS1	chr19:36322662	c.3325C>T	0	2	7	23	Kestilä M, *et al.* Mol Cell. 1998 Mar;1(4):575-82
Congenital nephrosis	NPHS1	chr19:36342512	c.121_122delCT	0	3	4	5	Kestilä M, *et al.* Mol Cell. 1998 Mar;1(4):575-82
Progressive myoclonic epilepsy	CSTB	chr21:45194163	c.218_219delTC	39	25	120	50	Bespalova IN, *et al.* Am J Med Genet. 1997 Sep 19;74(5):467-71
Progressive myoclonic epilepsy	CSTB	chr21:45194178	c.202C>T	36	27	98	50	Pennacchio LA, *et al.* Science. 1996 Mar 22;271(5256):1731-4
Progressive myoclonic epilepsy	CSTB	chr21:45194642	c.67-1G>C	53	27	33	45	Pennacchio LA, *et al.* Science. 1996 Mar 22;271(5256):1731-4
APECED	AIRE	chr21:45709656	c.769C>T	2	3	2	8	Nagamine K, *et al.* Nat Genet. 1997 Dec;17(4):393-8
APECED	AIRE	chr21:45711064	c.967_979del13[f]	0	1.8	14.6[g]	6.5	Finnish-German APECED Consortium. Nat Genet. 1997 Dec;17(4):399-403.
APECED	AIRE	chr21:45712943	c.1163_1164insA	8	0	15	22	Björses P, *et al.* Am J Hum Genet. 2000 Feb;66(2):378-92
X-linked retinoschisis	RS1	chrX:18665312	c.325G>C	38	35	97	149	Huopaniemi L, *et al.* Eur J Hum Genet. 1999 Apr;7(3):368-76
X-linked retinoschisis	RS1	chrX:18665416	c.221G>T	22	39	65	106	Huopaniemi L, *et al.* Eur J Hum Genet. 1999 Apr;7(3):368-76
X-linked retinoschisis	RS1	chrX:18665423	c.214G>A	21	41	56	101	Huopaniemi L, *et al.* Eur J Hum Genet. 1999 Apr;7(3):368-76
Salla type choroideremia	CHM	chrX:85133968	c.1609+2dupT	38	15	295	135	Sankila EM, *et al.* Nat Genet. 1992 May;1(2):109-13

All positions are given in genome build hg19 (GRCh37) [a] Mutation locus not targeted by the method. [b] Spans exons 10-13. Mean coverage of the exons is given. [c] Spans exons 7-8. Mean coverage of the exons is given. [d] Mean coverage across the deletion is given. [e] First 13 bp of the deletion is in CTR. [f] Mean coverage across the deletion is given. [g] First 4 bp of the deletion is in CTR.
